# Basal LAT-diacylglycerol-RasGRP1 Signals in T Cells Maintain TCRα Gene Expression

**DOI:** 10.1371/journal.pone.0025540

**Published:** 2011-09-26

**Authors:** Evan Markegard, Evan Trager, Chih-wen Ou Yang, Weiguo Zhang, Arthur Weiss, Jeroen P. Roose

**Affiliations:** 1 Department of Anatomy, University of California San Francisco, San Francisco, California, United States of America; 2 Department of Immunology, Duke University Medical Center, Durham, North Carolina, United States of America; 3 Department of Medicine, University of California San Francisco, San Francisco, California, United States of America; 4 Department of Microbiology and Immunology and Howard Hughes Medical Institute, University of California San Francisco, San Francisco, California, United States of America; 5 Rosalind Russell Medical Research Center for Arthritis, University of California San Francisco, San Francisco, California, United States of America; MRC National Institute for Medical Research, United Kingdom

## Abstract

In contrast to the well-characterized T cell receptor (TCR) signaling pathways that induce genes that drive T cell development or polarization of naïve CD4 T cells into the diverse T_H_1, T_H_2, T_H_17 and T_reg_ lineages, it is unclear what signals maintain specific gene expression in mature resting T cells. Resting T cells residing in peripheral lymphoid organs exhibit low-level constitutive signaling. Whereas tonic signals in B cells are known to be critical for survival, the roles of tonic signals in peripheral T cells are unknown. Here we demonstrate that constitutive signals in Jurkat T cell lines are transduced via the adapter molecule LAT and the Ras exchange factor RasGRP1 to maintain expression of *TCRα* mRNA and surface expression of the TCR/CD3 complex. Independent approaches of reducing basal activity through the LAT-diacylglycerol-RasGRP pathway led to reduced constitutive Ras-MEK-ERK signals and decreased *TCRα* mRNA and surface TCR expression in Jurkat cells. However, loss of TCR expression takes several days in these cell line experiments. In agreement with these in vitro approaches, inducible deletion of Lat in vivo results in reduced *TCRα* mRNA- and surface TCR- expression in a delayed temporal manner as well. Lastly, we demonstrate that loss of basal LAT-RasGRP signals appears to lead to silencing or repression of *TCRα* transcription. We postulate that basal LAT-diacylglycerol-RasGRP signals fulfill a regulatory function in peripheral T lymphocytes by maintaining proper gene expression programs.

## Introduction

The adaptor molecule LAT plays a crucial role in conducting intracellular signals following engagement of the T cell receptor (TCR) on thymocytes or peripheral T cells [1]. Phosphorylation of several conserved tyrosines in LAT provides a platform of binding sites for the SH2 domains of many other signaling molecules that connect receptor-proximal signals to downstream calcium and MAP kinase pathways [2]. Tyrosine-phosphorylated LAT recruits SLP-76 together with the Tec family kinase Itk, as well as phospholipase C γ1 (PLCγ1). Itk-mediated phosphorylation of phospholipase Cγ1 (PLCγ1) leads to its activation and to the generation of IP3 (inositol 1,4,5-trisphosphate) and DAG (diacylglycerol). IP3 binding to its receptors on the endoplasmic reticulum triggers the calcium pathway [3]. DAG can recruit PKC (protein kinase C) family members and RasGRPs (Ras guanyl nucleotide releasing protein) to the membrane [4] to increase RasGTP, which couples to the RAF-MEK-ERK kinase cascade to induce changes in gene expression. This includes increases in transcripts of *TCRα* and *TCRβ* [5] and the activation marker *CD69* [6]. These DAG-induced transcriptional events can be mimicked by treatment of cells with the synthetic DAG-analog PMA (phorbol 12-myristate 13-acetate). In addition to gene expression, these receptor-induced signals regulate the actin cytoskeleton [1]. For instance, SLP-76 can recruit the adapter Nck and the Rho/Rac GEF (guanine nucleotide exchange factor) Vav, which, together with other molecules, orchestrate cytoskeletal changes [2].

Both B- and T-lymphocytes also exhibit evidence of tonic antigen receptor signaling in the resting state, i.e. when antigen receptors are not “actively” triggered by their cognate antigens [7]. Freshly isolated thymocytes or peripheral T cells from lymph nodes demonstrate basal levels of TCRζ phosphorylation [8] and association of ZAP-70 with phospho-TCRζ [9], [10]. The precise origin and function of the signal(s) responsive to these receptor-proximal events is an area of debate. The role of tonic signaling in B lymphocytes has been more clearly delineated. B cells critically rely on expression of surface IgM for their survival [11]. The tonic signal has been suggested to result from BCR self-aggregation, raft-localization, and/or balances between BCR-associated kinases and phosphatases [7], and is, in part, mediated through a non-classical NFκB [12] and/or a PI3 kinase [13] pathway. The consequences of tonic TCR signals in naïve T cells is more controversial. Currently three non-exclusive roles have been proposed: one model suggests that the signal promotes survival [14]. Interestingly, inducible deletion of *TCRα* results in much more modest reduction in naïve CD4 T cell numbers (∼40% reduction in half-life) [15] compared to IgM removal from B cells [11]. The second and third models propose that tonic TCR signaling has an immuno-modulatory function, either enhancing [10] or blunting [16], [17] TCR responses to antigen.

In contrast to the biochemical signaling events and transcriptional responses that are known to occur when the TCR is fully stimulated by peptide-MHC [1], much less is known about the molecular and biochemical details of tonic signaling in T cells. We have previously demonstrated that tonic signals in cell lines and in *ex vivo* thymocytes repress the expression of a cluster of genes [18]. This cluster contains the *RAG* genes, just as in B cells [19], [20]. We characterized some of the responsible biochemical mechanisms and demonstrated that tonic ERK and Abl kinase activities depend on the adapter LAT to synergistically repress *RAG* gene expression [18]. Here we describe how tonic signals can also function in a reciprocal manner; that is, to maintain gene expression. We find that basal LAT-DAG-RasGRP1 dependent signals are critical to maintain normal expression levels of *TCRα* transcripts.

## Results

### Independent mutant Jurkat T cell lines with a defective basal Ras signaling pathway

**Figure 1:**
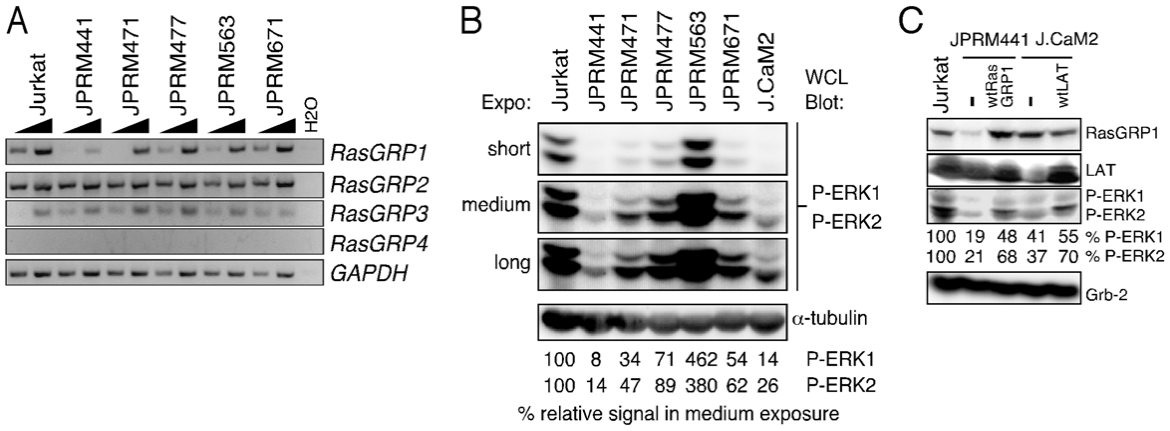
Loss of Basal Ras-ERK signaling in Jurkat-PMA-Response-Mutant (JPRM) clones. (A) RT-PCR analysis for *RasGRP-1, -2, -3, -4*, and *GAPDH* gene expression in wild type Jurkat T cells and a panel of mutant JPRM cell lines. Five-fold dilutions of cDNA input were used as template. (B) Western blot analysis of basal levels of phosphorylated ERK-1 and –2 in resting wild type Jurkat T cells, RasGRP1-deficient JPRM441 cells, LAT-deficient J.CaM2 cells, and 4 additional JPRM mutant cell lines. Equal loading is indicated by α-tubulin levels on the same blot. Relative levels of ERK-1 and –2 phosphorylation were calculated by normalizing for α-tubulin and set to 100% in the Jurkat sample. (C) Western blot analysis of RasGRP1 and LAT expression and basal levels of phosphorylated ERK-1 and –2 in the indicated resting cell types. All panels in figure 1 are representative examples of three independent experiments.

We previously identified a mutant clone, JPRM441, via a screen for mutant Jurkat T cell lines with defects in PMA-induced Ras activation and consequent CD69 upregulation (Figure S1A–C). We previously reported the signaling defects resulting from JPRM441's 90% reduction in RasGRP1 expression [21]. The same screen yielded additional clones. Complementation assays (Figure S1D) indicated that clones JPRM441, 471, 477, 563, and 671 are all unique. In contrast to JPRM441, these other 4 lines express normal levels of *RasGRP1, -2, and -3* mRNA (Figure 1A) as well as normal levels of RasGRP1 and PKCθ protein [21]. In addition to PMA-induced Ras activation, we analyzed basal activation of Ras as well as phosphorylation of ERK kinases that lie downstream of active Ras (RasGTP). These constitutive Ras-ERK signals were reduced in JPRM441, 471, 477, and 671 cells, comparable to the decreased basal signal we had previously reported for LAT-deficient J.CaM2 cells [18] (Figure 1B). In contrast, constitutive Ras-ERK signals were readily detectable in wild type Jurkat T cells and the JPRM563 line (Figure 1B and data not shown). The defects responsible for the impaired PMA-induced, or tonic, Ras signaling pathway in the JPRM471, 477, 563, and 671 lines are unknown. JPRM563 is unique in that transient expression of Ras with an activating mutation (H-RasG12V) does not induce CD69 upregulation (Figure S1E), demonstrating that a defect in JPRM563's pathway leading to CD69 lies downstream of Ras. All other lines, including JPRM441, demonstrated effective induction of CD69 expression driven by H-RasG12V, indicating that this oncogenic Ras allele can signal to the downstream RAF-MEK-ERK kinase cascade in these JPRM lines (Figure S1E).

We next analyzed Ras-ERK signaling in the basal state using the RasGRP1-deficient JPRM441 and LAT-deficient J.CaM2 cells, as well as lines stably reconstituted with RasGRP1 or LAT. Analysis of ERK phosphorylation revealed that this signal relies on the presence of LAT and RasGRP1 and that impaired tonic ERK phosphorylation was partially rescued in the wild type cDNA-reconstituted lines (Figure 1C).

### Loss of TCR/CD3 expression on mutant Jurkat T cell lines correlates with reduced tonic Ras-ERK signaling

**Figure 2:**
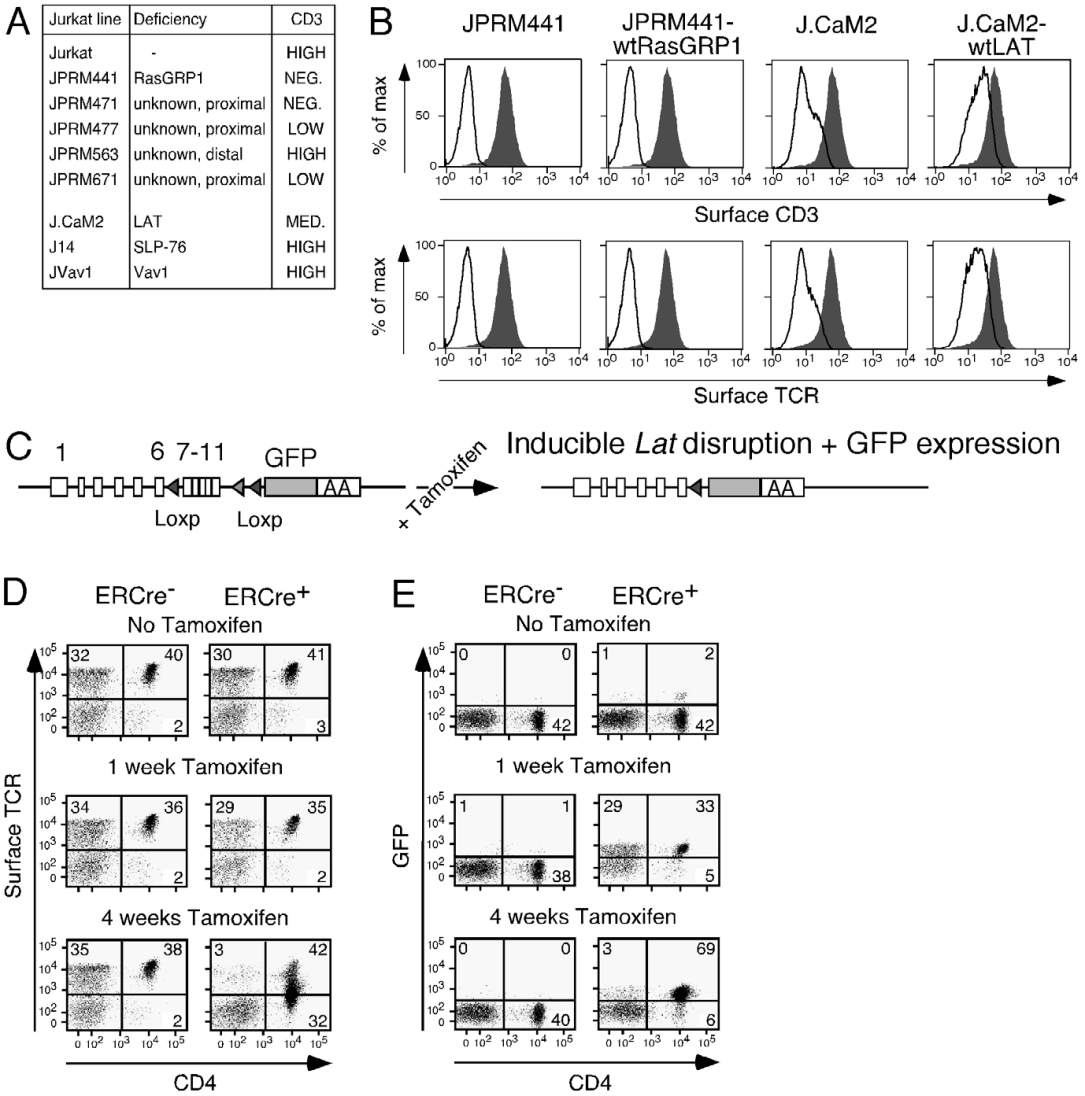
Decreased surface TCR/CD3 expression in Jurkat signaling mutant lines and primary T cells deleted for Lat. (A) Overview of the clonal cell lines used in this study, their identified deficiencies, and surface TCR/CD3 expression characteristics. (B) Surface CD3 expression by FACS analysis in the indicated cell lines. Solid grey histograms represent CD3 expression in wild type Jurkat cells. A representative example of three independent experiments. (C) Schematic of targeting approach that allows tamoxifen-inducible excision of exons 7–11 in Lat. (D, E) Dot plot FACS analyses of surface TCR expression levels and GFP expression against surface CD4 expression on the x-axis. Depicted are representative results of three independent experiments obtained with lymph node cells from ERCre^−^ Lat^f/−^ and ERCre^+^ Lat^f/−^ mice that either received no tamoxifen or tamoxifen on the first two days and subsequently every week. T cells were analyzed at 1 week or 4 weeks after the initial tamoxifen dose. Figures 2D and 2E are representative of findings with at least three individual mice per genotype.

A survey of surface CD3 expression on resting JPRM441 and other JPRM mutant lines revealed a surprising phenomenon: the observed defects in signaling proximal of Ras, but not distal of Ras, correlated with the loss of surface expression of the TCR/CD3 complex (Figure 2A). As we previously reported [18], LAT-deficient Jurkat cells (J.CaM2) [22] exhibited reduced TCR and CD3 surface expression (Figure 2A), as did some other clones generated by this J.CaM mutagenesis screen for Jurkat clones with defective TCR-induced calcium mobilization [23], [24]. A third screen for mutant Jurkat lines with defective TCR-induced CD69 upregulation utilizing retroviral insertion as a means of gene disruption also resulted in a subpopulation of CD3 negative cells [25]. Thus, loss of normal TCR/CD3 surface expression appears to be a common finding in these three screens designed to identify Jurkat T cell lines defective for intracellular signaling proteins.

To test if loss of LAT or RasGRP1 expression was responsible for loss of TCR/CD3 expression we examined stable reconstituted cell lines. Stable reconstitution of J.CaM2 cells with wild type LAT resulted in a relatively efficient restoration of CD3 surface expression (Figure 2B). In contrast, reconstitution of RasGRP1 expression did not have the same effect on CD3 expression in JPRM441 (Figure 2B). Additional JPRM441 clones with reconstituted RasGRP1 expression demonstrated the same resistance to reconstitution of CD3 expression (not shown). Analyses of expression of several other cell surface markers revealed that reconstitution of LAT or RasGRP1 resulted in a restoration of expression of some (e.g. CD38) but not of other (e.g. CD62L and CD2) cell surface markers (Figure S2). Given the recurrent correlation between lack of signaling molecules and TCR/CD3 expression in cell lines, we next focused our efforts on analyses of TCR/CD3 expression on primary T cells and on the mechanism that might underlie the reduced expression.

### In vivo removal of LAT results in reduced TCR expression in CD4 positive T cells

Until very recently it has been difficult to determine the *in vivo* function of tonic signaling and the involved signaling molecules in peripheral T cells, because T cell development is blocked in mice deficient for these molecules. LAT-deficient mice demonstrate an early and severe block in thymocyte development at the CD4/CD8 double negative stage [26], whilst RasGRP1-deficient thymocytes arrest at the CD4/CD8 double positive stage [27]. Both the Malissen and some of us recently constructed mouse models of inducible LAT deletion [28], [29]. We exploited our novel mouse model to examine the effects of LAT deletion in T cells on TCR expression utilizing induced *in vivo* LAT deletion via administration of tamoxifen (Figure 2C). Tamoxifen-induced activation of ERCre (Estrogen receptor-Cre recombinase fusion protein) results in efficient floxing of Lat's exons 7–11, loss of Lat protein after 3 days and induced GFP expression [30]. Here, ERCre^+^ or ERCre^−^ LAT^f/−^ mice were injected intraperitoneally with 100 µl of tamoxifen (10 mg/ml) on two consecutive days and were then treated with tamoxifen weekly. Whereas surface TCR expression on CD4^+^ T cells was unaltered 1 week after tamoxifen treatment, we observed a significant reduction in expression 4 weeks after treatment (Figure 2D). Note that the treatment also resulted in a relative increase in the percentage of CD4 positive cells. The reduction in TCRβ expression was specific and not induced by tamoxifen in ERCre-negative mice that were analyzed as controls (Figure 2D). Based on GFP expression at 4 weeks, the recombination of loxP sites in the targeted *Lat* locus occurred with a ∼90% efficiency in CD4^+^ T cells (Figure 2E).

### Impaired tonic Ras-ERK-CD69 signaling correlates with loss of *TCRα* transcripts

Previous work demonstrated that PMA directly induces increased *TCRα* and *TCRβ* expression in T cells, that is, when protein synthesis is blocked by cycloheximide [5]. We formerly reported that LAT-ERK/Abl signals synergize to suppress expression of a specific cluster of genes including the *RAG* genes; *RAG1* and *RAG2* mRNA expression is aberrantly high in LAT-deficient J.CaM2 cells [18]. We hypothesized that similar tonic (DAG) signals from LAT and via RasGRP1 might function to maintain the expression levels of the direct target genes, *TCRα* and/or *TCRβ* in the basal state.

**Figure 3:**
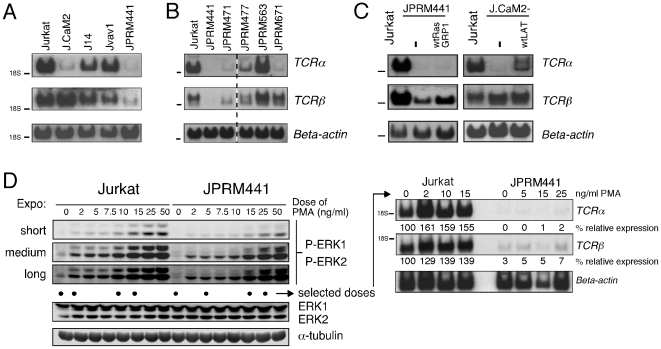
Loss of *TCRα* transcripts in mutant Jurkat cell lines with impaired tonic Ras-ERK signaling. (A, B) Northern blot analysis of transcript levels of *TCRα* and *TCRβ* in a panel of resting wild type and mutant Jurkat cell lines. *Beta-actin* mRNA levels were assessed as a control for loading. (C) Northern blot analysis of transcript levels of the *TCRα*, *TCRβ*, and *Beta-actin* in Jurkat, JPRM441, J.CaM2, and the two mutant lines stably reconstituted with RasGRP1 or LAT cDNA. (D) Western blot analysis of Jurkat and JPRM441 samples stimulated for 10 minutes with the indicated doses of PMA. Levels of ERK-1 and –2 phosphorylation and α-tubulin were determined on the same blot. Northern blot analysis of transcript levels of *TCRα* and *TCRβ* in wild type Jurkat and RasGRP1 deficient JPRM441 left unstimulated or stimulated with 2, 10, and 15, or for JPRM441 with 5, 15, and 25 ng/ml PMA for 20 hours, respectively. Percentages of relative expression were determined by normalizing for *Beta-actin* expression. The unstimulated Jurkat sample was set at 100%. All panels in figure 3 are representative examples of three independent experiments.

Northern blot analyses demonstrated a marked reduction of *TCRα* transcript levels when LAT or RasGRP1 are absent and a more moderate decrease in lines without SLP-76 (J14) or Vav1 (Jvav1) (Figure 3A). Similarly, levels of *TCRα* mRNA were reduced in JPRM471, JPRM477, and JPRM671 (Figure 3B). Transcript levels of *TCRβ* were less affected by the absence of the various signaling molecules in the basal state (Figure 3A and B). The specific pattern of *TCRα* transcript levels in the various mutant JPRM cell lines followed the pattern of basal ERK phosphorylation; the more moderate defect in basal P-ERK in JPRM477 correlated with a more moderate reduction in *TCRα* mRNA levels, while *TCRα* transcripts were comparable to wild type control in the JPRM563 line, which has normal basal ERK phosphorylation (Figure 3B and 1B).

Stable reconstitution of J.CaM2 cells with wild type LAT resulted in substantial rescue of *TCRα* mRNA expression. Restoration of RasGRP1 expression did not have the same effect on either *TCRα* or *TCRβ* expression in JPRM441 (Figure 3C). The failure to rescue *TCRα* or *TCRβ* expression was not a reflection of one particular cell line (clone 208); two independent stable clones (clones 240 and 247 [21]) with restored RasGRP1 expression demonstrated the same defective pattern in TCR restoration (data not shown). We were puzzled by the lack of restoration of *TCRα* and *TCRβ* mRNA expression in RasGRP1-reconstituted JPRM441 cells. We wondered whether there are any Ras–dependent signals that can restore expression. Deliberate triggering of the Ras-ERK signaling pathway in these cells via high doses of PMA did not lead to increased expression of *TCRα* or *TCRβ* mRNA (Figure 3D). In contrast, we previously showed that PMA triggers robust induction of *TCRα* expression in J.CaM2 cells [18]. Similarly, transient overexpression of RasGRP1 or oncogenic H-RasG12V in JPRM441 cells did not restore *TCRα* and *TCRβ* mRNA or surface CD3 expression in JPRM441 cells. Only co-transfection of both TCR*α*- and TCR*β*- expression plasmids in these cells effectively rescued CD3 expression (Figure S3). These results indicate that tonic signals from LAT and via RasGRP1 are important in maintaining *TCRα* gene expression. However, it also appears that, while *TCRα* expression was substantially repressed in LAT-deficient J.CaM2 cells, *TCRα* is more irreversibly repressed in RasGRP1-deficient JPRM441, perhaps even silenced.

### In vivo deletion of LAT results in time-dependent loss of *TCRα* transcripts in CD4 positive T cells

Analyses of mice with deletion of LAT or expression of mutant LAT^Y136F^ in peripheral T cells revealed surprising findings; these mice developed the same lymphoproliferative disorder as when LAT^Y136F^ was introduced in the germline, resulting in thymocytes (and T cells) expressing LAT^Y136F^ protein [28], [30], [31], [32]. Thus, LAT possesses an unexpected control function in peripheral T cells in addition to its function during thymocyte selection [33]. As a result of loss of this control, LAT-deleted CD4^+^ T cells become CD44^+^CD62L^lo^ by 3–4 weeks, a phenotype of activated/memory T cells and these CD4^+^ T cells produce high levels of intracellular IL-4 [30]. We hypothesized that part of this control function through LAT might reflect maintaining correct gene expression in resting peripheral CD4 T cells and that loss of LAT might lead to loss of *TCRα* mRNA expression, similar to what we observed in the LAT-deficient J.CaM2 line (Figure 3).

**Figure 4:**
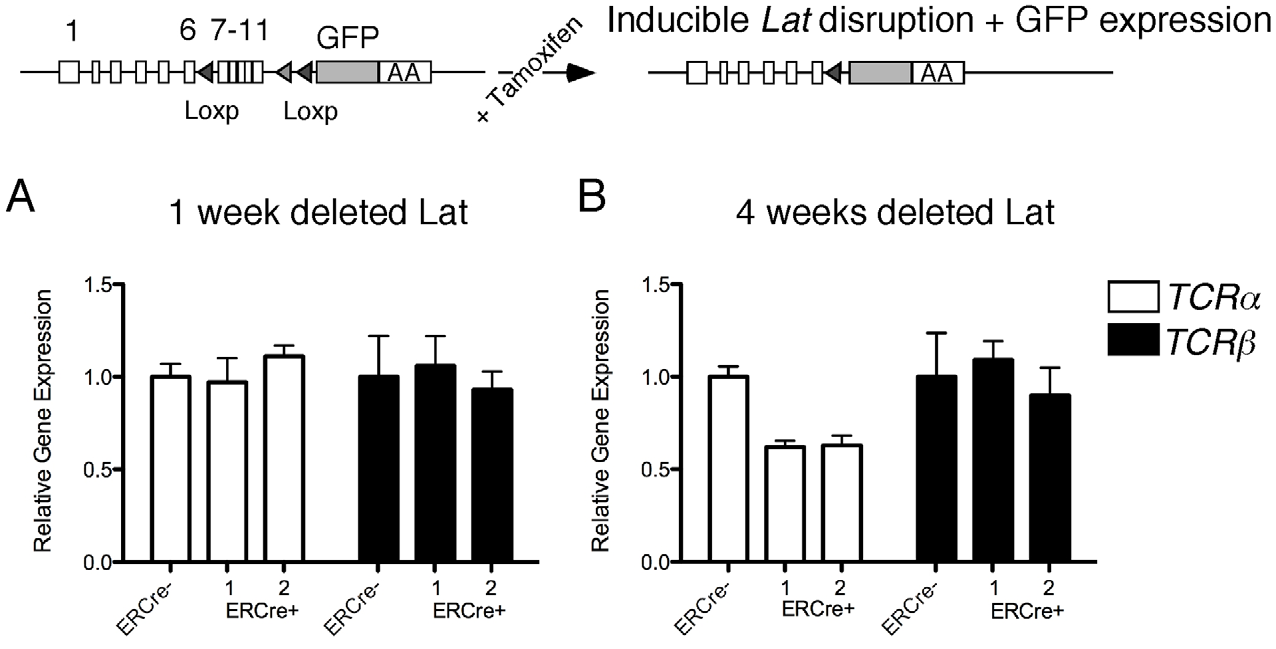
LAT-deletion in primary CD4^+^ T cells results in time-dependent loss of *TCRα* transcripts. (A, B) Taqman PCR analysis of *TCRα* and *TCRβ* mRNA expression levels in sorted CD4^+^CD44^low^ T cells from two ERCre^+^ Lat^f/−^ mice and one control ERCre^−^ Lat^f/−^ mouse. Error bars represent taqman triplicates. The results are a representative example of two independent experiments.

To test this hypothesis we isolated RNA from MACS-purified CD4^+^ T cells with low levels of CD44 expression (CD44^low^) that were depleted from LAT expression for either 1 week or for 4 weeks. Taqman PCR analyses on two individual mice 1 week after tamoxifen treatment revealed that *TCRα* and *TCRβ* expression levels were unaltered and comparable to the ERCre-negative control (Figure 4A). However, when LAT was deleted for 4 weeks, CD4^+^CD44^lo^ T cells exhibited decreased amounts transcripts of *TCRα*, but preservation of *TCRβ* (Figure 4B). Thus, *TCRα* mRNA is maintained in a LAT-dependent manner in both Jurkat T cells as well as in naïve primary CD4^+^CD44^lo^ T cells. Loss of LAT does not lead to an immediate reduction of *TCRα* transcript levels but, rather, appears to take several weeks.

### Tonic DAG-RasGRP1 signals maintain *TCRα*, and *TCRβ* transcripts

**Figure 5:**
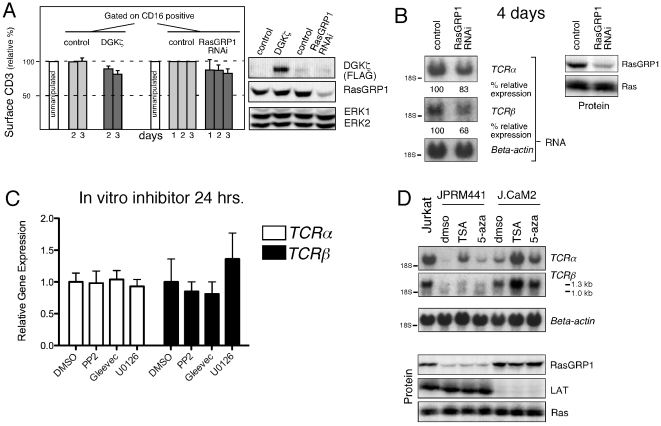
Repression of *TCRα* expression by limiting DAG-RasGRP1 signals requires several days and can be reversed by TSA or 5-aza inhibitors. (A) Bargraph representing mean fluorescence signals for surface expression of CD3 on wild type Jurkat cells transfected with vector control, RasGRP1 RNAi, or DGKζ expression constructs, all together with a CD16 expression construct as marker. Surface CD3 expression was determined on CD16 positive cells, at 1, 2, or 3 days following transfection. Expression of CD3 on unmanipulated cells was arbitrarily set at 100%. Means and standard errors of 3 independent experiments are depicted. Western blot analysis of a representative experiment, analyzing unsorted cells for the indicated protein expression 3 days after transfection, indicates the level of FLAG-tagged DGKζ expression and the degree of RasGRP1 knockdown. (B) Northern blot analysis of transcript levels of the indicated TCR components and Western blot analyses of the indicated protein expression levels examining Jurkat T cells 4 days after transient transfection with the indicated RNAi or control construct. (C) Taqman PCR analysis of *TCRα* and *TCRβ* mRNA expression levels in sorted CD4^+^CD44^low^ T cells from wild type mice treated for 24 hours with the indicated inhibitors in the absence of stimuli. Error bars represent taqman triplicates. (D) Northern blot analysis of transcript levels of the indicated TCR components and Western blot analyses of the indicated protein expression levels examining the indicated cell lines treated for 20 hours with Trichostatin A (TSA) or 5-azacytidine (5-aza). Of note, combined treatment of TSA and 5-aza was too toxic to obtain good quality RNA. Panels 5B–5C are representative examples of three independent experiments.

Currently there are no mouse models available to delete Rasgrp1 in primary T cells in an inducible manner. We therefore took alternative approaches to substantiate a potential role of RasGRP in tonic signaling leading to regulation of *TCRα* and *TCRβ* in addition to the chemically mutagenized lines (Figure 3). Diacylglycerol kinases (DGK) convert DAG to phosphatidic acid and thereby limit DAG-dependent downstream signals [34]. Both DGKζ overexpression or introduction of a RasGRP1 RNAi construct, resulted in a small, but reproducible, reduction in surface CD3 expression that required several days to have an effect (Figure 5A). RasGRP1 RNAi also resulted in a modest reduction of *TCRα* and *TCRβ* mRNA when RasGRP1 expression was suppressed for 4 days (Figure 5B). No effects were observed with shorter periods of RasGRP1 reduction (data not shown). While modest, these effects were remarkable given the fact that the results in Figure 5A are dependent on protein turnover, whereas in Figure 5B only a portion of the population of wild type Jurkat cells are transfected with the RNAi construct (∼40% transfected, data not shown).

These cell line approaches support the role of basal DAG-RasGRP1 signals regulating surface CD3 levels and *TCRα* and *TCRβ* mRNA expression in Jurkat cells. The results also suggest that the decrease of *TCRα* and *TCRβ* mRNA expression is a consequence of limiting DAG-RasGRP1 signals and do not occur immediately. We previously demonstrated that tonic signals repress *RAG1* gene expression in thymocytes and that blockade of this signal for a relatively short 24 hr.-period via addition of the Src kinase inhibitor PP2, the Abl kinase inhibitor Gleevec or the MEK kinase inhibitor U-0126 resulted in increased *RAG1* gene re-expression in cultured thymocytes [18]. Here we utilized the same inhibitors to test the effects of short-term inhibition of tonic signals on the expression levels of *TCRα* and *TCRβ* in primary T cells. We purified CD4^+^CD44^lo^ T cells from lymph node and cultured them without stimuli for 24 hours *in vitro* in the presence of these inhibitors or DMSO as vehicle-control. Taqman PCR analyses revealed that levels of *TCRα* and *TCRβ* transcripts remained relatively constant and were unaffected by 24-hr inhibition of the Src-, Abl-, or MEK- kinases (Figure 5C).

The fact that changes in *TCRα* transcripts as a function of loss of tonic signaling in cell lines and primary T cells took a certain period of time to manifest (Figures 4 and 5) and the fact that *TCRα* and *TCRβ* expression was not rescued by reconstituted RasGRP1 expression in JPRM441 (Figure 3) prompted us to test if gene silencing of TCR transcripts in JPRM cells could be involved. Hallmarks of silent chromatin are unacetylated histone proteins and a high frequency of methylated CpG dinucleotides [35]. Histone acetyl groups can be removed by HDACs [36]. Trichostatin A (TSA) is a compound that inhibits class I and II HDACs [37]. Methylation of CpG occurs via DNA methyltransferases (DNMTs) while demethylation occurs during DNA replication [36]. As a consequence, *de novo* methylation during DNA replication can be blocked by addition of 5-aza-2′deoxycytidine (5-aza). Treatment of JPRM441 with TSA resulted in re-expression of *TCRα* mRNA and a more modest increase in *TCRβ* expression (Figure 5D). Restoration of *TCRα* expression was more evident in J.CaM2 cells and TSA also induced increased levels of *TCRβ* transcripts in these cells. Addition of 5-aza resulted in a similar pattern of re-expression with more moderate effects. Of note, increased expression of *TCRα* and *TCRβ* induced by TSA or 5-aza was not a result of rescue of RasGRP1 or LAT protein expression (Figure 5D).

## Discussion

Here we have demonstrated that basal LAT-DAG-RasGRP1 signals function to maintain *TCRα* gene expression in Jurkat T cell lines and in a mouse model of inducible-Lat deletion. RasGRP1 is positioned downstream of LAT in the traditional signaling cascade that is induced when the TCR is fully stimulated by peptide-MHC [1]. Unexpectedly, expression of *TCRα* mRNA in the basal state is more strongly repressed in RasGRP1-deficient JPRM441 cells than in LAT-deficient J.CaM2 cells. Also, *TCRβ* expression levels are reduced in RasGRP1-deficient Jurkat cells and in Jurkat cells with RNAi-driven reduction of RasGRP1 expression for 4 days, but not in LAT-deficient Jurkat cells (permanently in J.CaM2) or in primary T cells without Lat (for 4 weeks). It appears that these loci are less repressed and still partially accessible for transcription factor regulation in J.CaM2 cells, i.e. so that restoration of LAT expression or stimulation with PMA leads to re-expression of *TCRα*, whereas in JPRM441 cells this did not occur. In agreement, TSA- and 5-aza treatments had a more dramatic effect on *TCRα* re-expression in J.CaM2 cells. What explains the discordant TCR chain expression pattern in these two signaling defective cell lines and in primary T cells? It is possible that there are additional defective molecules in JPRM441 cell line associated with the random EMS mutagenesis that affect expression of *TCRα* and *TCRβ* in the basal state. An additional explanation for the difference between JPRM441 and J.CaM2 cells is that there are functional differences between the lost basal Ras-ERK signals when RasGRP1 or LAT is absent. Lack of the required mouse model for inducible Rasgrp1 deletion and lack of capability to culture viable, primary T cells for long periods of time with inhibitors prevent us from addressing the difference between Rasgrp1 and Lat in primary T cells at this point.

Expression of RasGRP1 or an active allele of Ras (H-RasG12V) did not result in rescue of TCR expression. However, reconstitution of JPRM441 cells with RasGRP1 did result in restoration of PMA-induced CD69 expression [21] and expression of H-RasG12V in JPRM441 cells results in CD69 induction as well. Thus, while all three genes (*TCRα*, *TCRβ*, and *CD69*) are PMA-responsive [5], [6], mechanisms of gene regulation in the basal state appear distinct between CD69 and the *TCRα* and *TCRβ* genes. In concurrence, CD69's chromatin appears equally accessible in thymocytes expressing either low or high levels of surface CD69 [38]. In addition, even though both *TCRα* and *TCRβ* genes are PMA-responsive, tonic LAT-DAG-RasGRP1 signals seem to play a more important role in the regulation of *TCRα* expression in the basal state.

Various mouse models carrying hypomorphic alleles of signaling molecules in the germline demonstrate interesting immune phenotypes. For instance, an SKG allele of ZAP-70 with reduced binding-affinity for phospho-TCRζ leads to autoimmune arthritis in mice [39]. As discussed, germline mutation of the single tyrosine in LAT (LAT^Y136F^) also results in an autoimmune phenotype, accompanied with hyperproliferative lymphocytes of a T_H_2 type, producing IL-4 cytokine [31], [32]. It has been suggested that these immune phenotypes are caused by altered thymocyte selection: selection of TCR's that display a high affinity for self might be a way of compensation for the impaired intracellular signaling molecules. These T cells bearing self-reactive TCRs often lead to autoimmune syndromes in mouse models. Results obtained with recent mouse models of inducible LAT deletion [28], [29] suggest that additional regulatory mechanisms that operate in peripheral T cells play a role as well. Unexpectedly, induced deletion of LAT or expression of LAT^Y136F^ in peripheral T cells leads to the same lymphoproliferative syndrome as when LAT is mutated in thymocytes [28], [30], [31], [32]. These findings challenge the current dogma that these autoimmune phenotypes are exclusively caused by an altered T cell repertoire. Instead, it appears that (tonic signals through) LAT possesses an unexpected surveillance function in peripheral T cells [33]. We observed that inducible removal of Lat leads to reduced *TCRα* mRNA expression levels and loss of TCR surface expression on roughly 40% of the CD4 T cells within 4 weeks. We postulate that this will lead to a further loss of constitutive TCR signals. E.g., loss of optimal surface TCR expression may lead to less tonic signal coming from self-MHC/TCR interactions [7], resulting in lower basal levels of the normally observed TCRζ phosphorylation [8] and basal association of ZAP-70 with phospho-TCRζ [9], [10]. The results presented here offer a potential hypothesis for the mechanism underlying the interesting immune phenotypes observed in mice with inducible Lat deletion [28], [29] that can be explored in the future. We postulate that tonic signals in peripheral T cells through LAT contribute to epigenetic regulation of *TCRα* transcripts and possibly other gene expression profiles and, as such, fulfills a controlling function of T cell activity in the basal state.

## Materials and Methods

### Cell lines, cell purification, stimulations, inhibitors, FACS analysis, transfections, and RNAi

Jurkat T cells, derived JPRM, J.CaM2. J14, and JVav1 mutant lines were grown and analyzed for expression of cell surface markers by FACS as described before [18], [21]. Surface expression of TCR was determined using an anti TCRα/β antibody (Caltag, MHAB01). Purification of CD4 T cells was performed by negative depletion and MACS (magnetic cell sorting) as described before [40]. In short, single cell suspensions were obtained, filtered, and stained with a cocktail of biotinylated antibodies against CD117, Ly6G, MHC class II, CD19, CD24, CD8. Cells were washed and incubated with Streptavidin beads (Miltenyi Biotec) and run over a depletion column avoiding saturation of the column. Cells were seeded at 1 million/ml in RPMI supplemented with 15% fetal calf serum. Details of stimulations, inhibitors, transfections, FACS analysis, and RasGRP1 RNAi were previously described [18], [21]. TSA was added at 20 nM, 5-aza at 2.5 µM. CD4^+^ T cells from ERCre^+^ or ERCre^−^ LAT^f/−^ mice treated with tamoxifen were purified by using EasySep mouse CD4+ T cell Pre-Enrichment kit (Stemcell). The resulting cells demonstrated a 95%, 94%, and 96% CD4+ T cell purify for ERCre+1, ERCre+ 2, and ERCre−. All animal work has been conducted according to relevant national and international guidelines, described in our animal protocol “Ras Signal Transduction in Lymphocytes and Cancer”, which was approved by the INSTITUTIONAL ANIMAL CARE AND USE COMMITTEE (IACUC) at UCSF University California San Francisco, protocol number AN084051-01A. Additional animal work was performed at Duke University and procedures were approved by the Duke University IACUC. The protocol number for this study is A216-08-08.

### Generation of EMS mutants

Jurkat PMA Response Mutants (JPRM) were generated by mutagenesis of 100×10^6^ Jurkat E6-1-clone1 cells with 200 µg/ml EMS (Sigma) for 24 hr., and subsequent selection based on a defect in PMA-induced CD69 expression as described [21].

### Plasmids

RasGRP1, H-RasG12V, PKCθ(AE), CD16/7, and GFP expression constructs were described before [21], [41]. Expression constructs for TCRα and TCRβ were previously described [42], [43]. Expression of these cDNAs is driven by a constitutive EF1-α promoter. An expression plasmid for DGKζ was generously provided by Gary Koretzky [44].

### Western blot analysis

Expression levels of various proteins were determined and quantitated by Western blot analysis of 1% NP40 lysates and analyzed by quantitative luminescence on a Kodak Image Station as described before [21]. Antibodies to phospho-ERK, α-tubulin, RasGRP1, Grb-2, FLAG, Ras, and ERK proteins were previously described [18], [21]. Antibodies to LAT (Abcam) were purchased.

### RNA isolation, Northern blot analysis, RT PCR, and Taqman

RNA was isolated and TCRα and beta-actin Northern blot hybridizations were carried out and quantified as described in detail before [18]. For TCRβ expression analysis, a 350 bp BglII-StuI fragment was used. For RT-PCRs, first strand cDNA was produced and RasGRP1-specific, RasGRP2-specific, RasGRP3-specific, RasGRP4-specific, and HPRT-specific PCRs were carried out, all as described before [21]. For TaqMan Real-Time PCR, RNA was isolated using Trizol Reagent (Invitrogen, Carlsbad, CA) and Chloroform (Sigma-Aldrich, St, Louis, MO) extraction followed by Rneasy MiniElute Cleanup Kit (Qiagen, Gaitherburg, MD) following manufactures recommendations. 2 µg of RNA was converted to cDNA by using SuperScript First-Strand Synthesis System for RT-PCR (Invitrogen, Carlsbad, CA) following manufactures recommendations for random primer procedure. TaqMan Real-Time PCR was done using SensiMix II Probe Kit (Bioline, Tauton, MA) following manufacture's recommendations. T-cell receptor alpha (Tcrα), and T-cell receptor beta (Tcrβ) mRNA was expressed relative to β-actin (Actb) using the 2^−ΔCT^ method. The Mastercycler ep realplex^2^ system (Eppendorf, Hauppauge, NY) was used for all TaqMan assays following manufactures recommendations. N = 3 for all assays. Primer and probe sequences are as follows. TCRα F5′-CAAGCTTCACCTGCCAAGAT-3′, R 5′-AAATCCGGCTACTTTCAGCA-3′, Probe 5′-CGTTCCCTGTGATGCCACGTTG-3′. TCRβ, F 5′-GCACAATCCTCGAAACCACT-3′, R 5′-CCCTGATGATAGGATGCTGAA-3′, Probe 5′-GTGGCCAGAGGGCTCACCCA-3′. βActin Mm01205647_g1 (Applied Biosystems, Foster City, CA).

## Supporting Information

Figure S1 **Generation and analysis of JPRM mutant Jurkat clones.** (S1A) Screening strategy to obtain 125 mutant Jurkat clones defective in PMA-induced CD69 upregulation. EMS (methanesulfonic acid ethyl ester) was used to introduce random point mutations into the Jurkat genome. Jurkat T cells are haplo-insufficient for many genes making it possible to generate a functional mutant line by targeting only one allele. (S1B) FACS analysis of CD69 expression on PMA-stimulated wildtype Jurkat T cells compared to PMA-stimulated pools of mutant cells of subsequent rounds of negative selection. (S1C) 23 clonal lines of the JPRM441 RasGRP1-deficient JPRM cell type belonged to a larger selection of a total of 45 lines, all with severe defects in CD69 upregulation. FACS analysis comparing CD69 expression on unstimulated and 20 hrs.-PMA stimulated wildtype Jurkat cells and the indicated, clonal, mutant JPRM cell lines. (S1D) Typical representation of a complementation assay using polyethylene glycol (PEG)-induced cell fusion. The indicated cell lines in three columns and three rows were individually labeled with either CFSE or PHK26. Cells were fused making all CFSE (green) to PHK26 (red) combinations of each cell line to itself or to a different line. 8 hr later, the resultant cell populations were stimulated with PMA. FACS dot plots demonstrate the CFSE+PHK25+ cells that were analyzed for induced CD69 expression on the right, 16 hr after stimulation. (S1E) FACS analysis of GFP and induced CD69 expression on Jurkat, JPRM441, JPRM471, JPRM477, JPRM563, and JPRM671 cells, 40 hr after cotransfection of 10 µg GFP with 10 µg vector or 10 µg active H-RasG12V. Numbers indicate the percentages of live cells in each quadrant. Similar patterns were obtained 20 hrs. after transfection.
(JPG)

Figure S2 **Survey of cell surface marker expression in RasGRP1- and LAT-reconstituted JPRM441 and J.CaM2 mutant lines.** FACS analysis of surface markers on JPRM441 and J.CaM2 mutant lines. Note that expression of CD3 and TCR was restored to some extend in LAT reconstituted J.CaM2 cells (J.CaM2-wtLAT) but not rescued in JPRM441-wtRasGRP1. In contrast, CD38 expression is restored in both reconstituted lines. CD2 and CD62L are expressed at lower levels in JPRM441 and J.CaM2 and are not restored in either JPRM441-wtRasGRP1 or J.CaM2-wtLAT lines. Of note, we have subcloned both the J.CaM2 and the J.CaM2-wtLAT line through limiting dilution on several occasions. In all subclones of J.CaM2 we consistently detect a small shoulder of higher CD3- or TCR-expression, but never the uniform levels of increased CD3 and TCR expression we observe for J.CaM2-wtLAT.
(JPG)

Figure S3 **Impaired CD3 expression on RasGRP1-deficent JPRM441 is only restored upon plasmid-derived expression of TCRα and TCRβ.** FACS analysis of GFP and induced CD3 expression on JPRM441 cells, 40 hr after cotransfection of 10 µg GFP with either 10 µg vector, 10 µg active H-RasG12V, 10 µg RasGRP1 (on two different vector backbones), 10 µg TCRα, or 10 µg TCRα+10 µg TCRβ expression plasmids. Numbers indicate the percentages of live cells in each quadrant.
(JPG)
