# Supplementary figures and images for: Basal LAT-diacylglycerol-RasGRP1 Signals in T Cells Maintain TCRα Gene Expression
Source: PLoS One. 2011 Sep 26;6(9):e25540. doi: 10.1371/journal.pone.0025540 (PMC3180458; doi:10.1371/journal.pone.0025540)

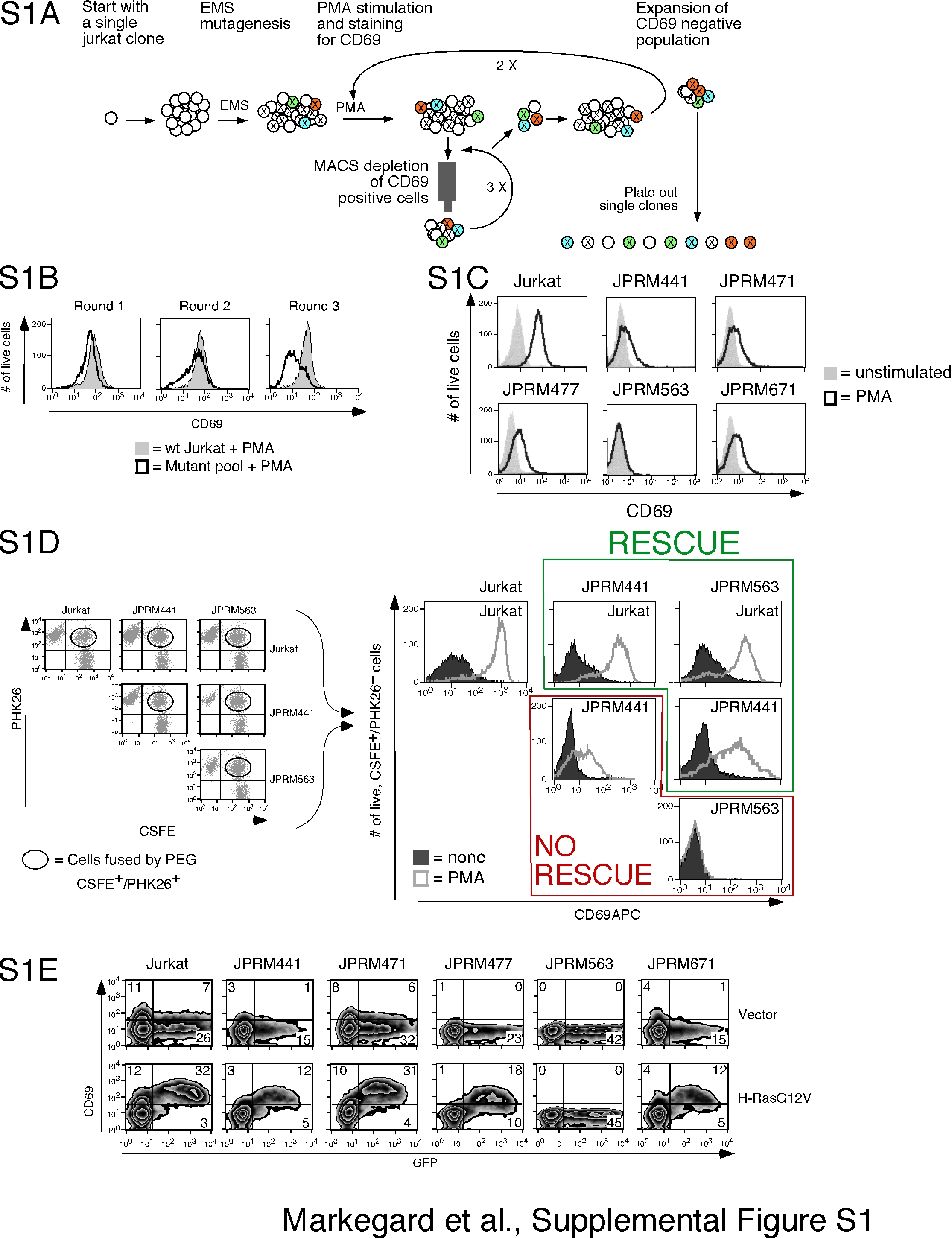

Supplement: Figure S1 — Generation and analysis of JPRM mutant Jurkat clones. (S1A) Screening strategy to obtain 125 mutant Jurkat clones defective in PMA-induced CD69 upregulation. EMS (methanesulfonic acid ethyl ester) was used to introduce random point mutations into the Jurkat genome. Jurkat T cells are haplo-insufficient for many genes making it possible to generate a functional mutant line by targeting only one allele. (S1B) FACS analysis of CD69 expression on PMA-stimulated wildtype Jurkat T cells compared to PMA-stimulated pools of mutant cells of subsequent rounds of negative selection. (S1C) 23 clonal lines of the JPRM441 RasGRP1-deficient JPRM cell type belonged to a larger selection of a total of 45 lines, all with severe defects in CD69 upregulation. FACS analysis comparing CD69 expression on unstimulated and 20 hrs.-PMA stimulated wildtype Jurkat cells and the indicated, clonal, mutant JPRM cell lines. (S1D) Typical representation of a complementation assay using polyethylene glycol (PEG)-induced cell fusion. The indicated cell lines in three columns and three rows were individually labeled with either CFSE or PHK26. Cells were fused making all CFSE (green) to PHK26 (red) combinations of each cell line to itself or to a different line. 8 hr later, the resultant cell populations were stimulated with PMA. FACS dot plots demonstrate the CFSE+PHK25+ cells that were analyzed for induced CD69 expression on the right, 16 hr after stimulation. (S1E) FACS analysis of GFP and induced CD69 expression on Jurkat, JPRM441, JPRM471, JPRM477, JPRM563, and JPRM671 cells, 40 hr after cotransfection of 10 µg GFP with 10 µg vector or 10 µg active H-RasG12V. Numbers indicate the percentages of live cells in each quadrant. Similar patterns were obtained 20 hrs. after transfection. (JPG) [file pone.0025540.s001.jpg]

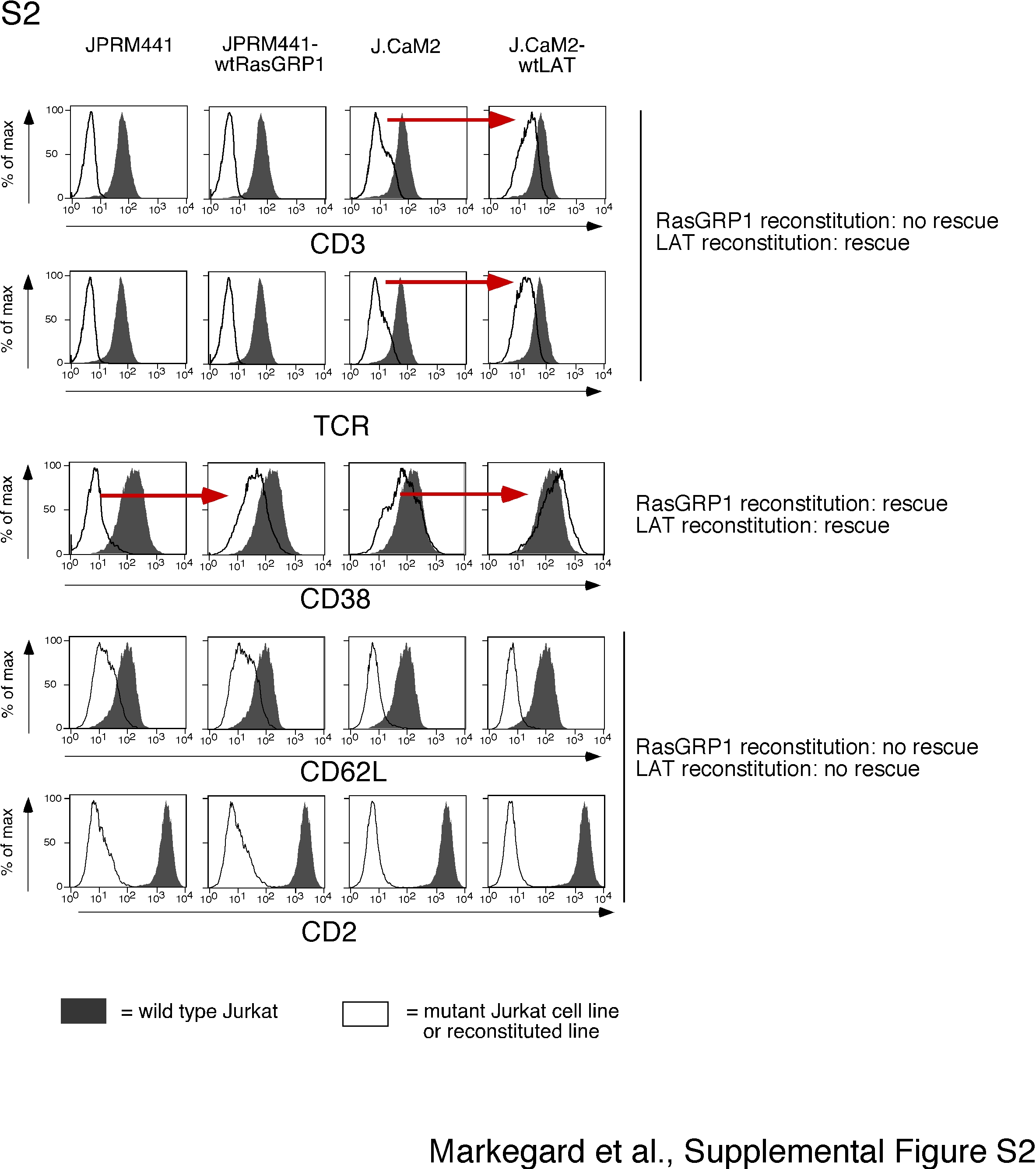

Supplement: Figure S2 — Survey of cell surface marker expression in RasGRP1- and LAT-reconstituted JPRM441 and J.CaM2 mutant lines. FACS analysis of surface markers on JPRM441 and J.CaM2 mutant lines. Note that expression of CD3 and TCR was restored to some extend in LAT reconstituted J.CaM2 cells (J.CaM2-wtLAT) but not rescued in JPRM441-wtRasGRP1. In contrast, CD38 expression is restored in both reconstituted lines. CD2 and CD62L are expressed at lower levels in JPRM441 and J.CaM2 and are not restored in either JPRM441-wtRasGRP1 or J.CaM2-wtLAT lines. Of note, we have subcloned both the J.CaM2 and the J.CaM2-wtLAT line through limiting dilution on several occasions. In all subclones of J.CaM2 we consistently detect a small shoulder of higher CD3- or TCR-expression, but never the uniform levels of increased CD3 and TCR expression we observe for J.CaM2-wtLAT. (JPG) [file pone.0025540.s002.jpg]

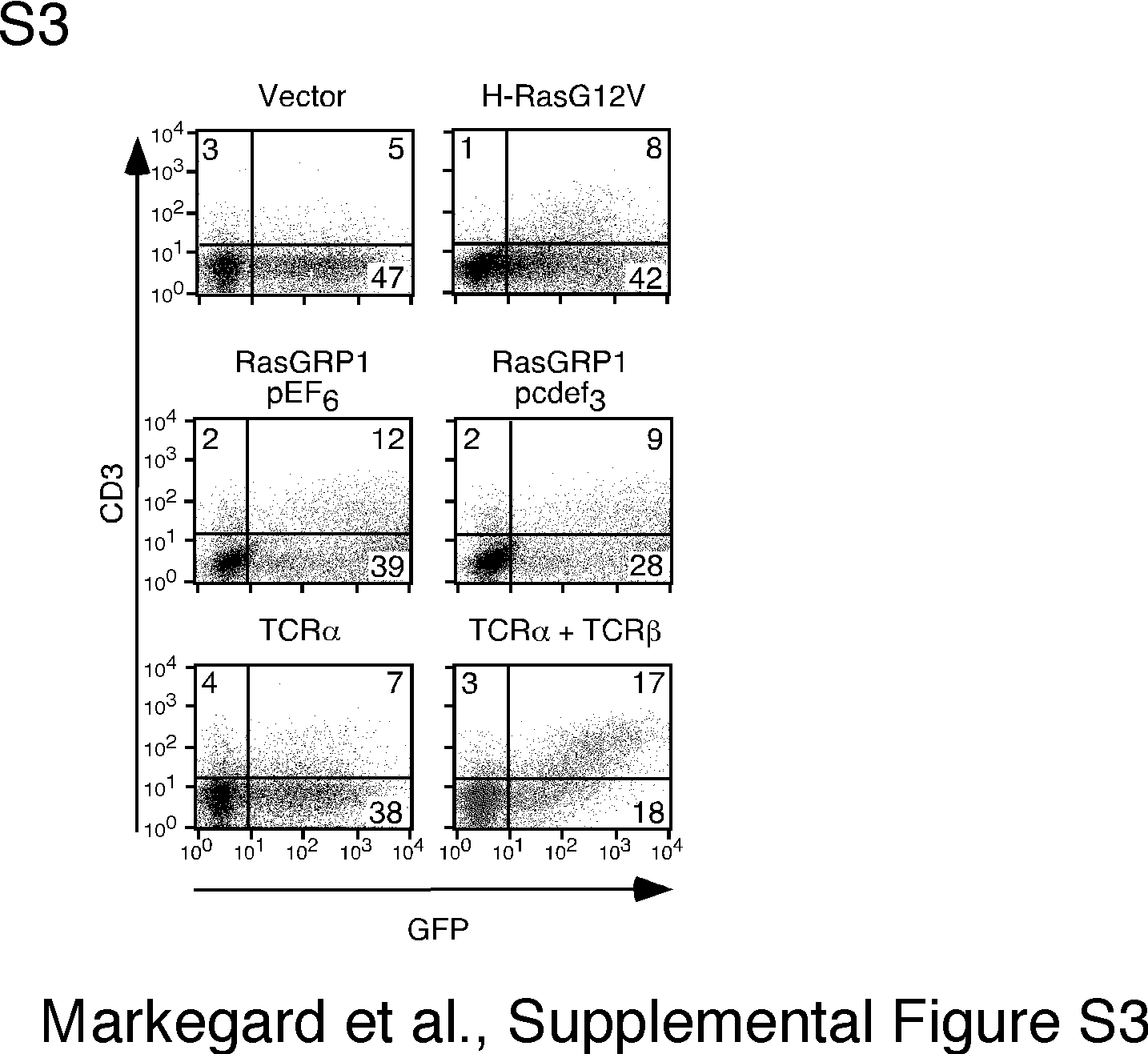

Supplement: Figure S3 — Impaired CD3 expression on RasGRP1-deficent JPRM441 is only restored upon plasmid-derived expression of TCRα and TCRβ. FACS analysis of GFP and induced CD3 expression on JPRM441 cells, 40 hr after cotransfection of 10 µg GFP with either 10 µg vector, 10 µg active H-RasG12V, 10 µg RasGRP1 (on two different vector backbones), 10 µg TCRα, or 10 µg TCRα+10 µg TCRβ expression plasmids. Numbers indicate the percentages of live cells in each quadrant. (JPG) [file pone.0025540.s003.jpg]
